# An Atlas of Anatomical Plates of the Human Body, Accompanied with Descriptions in Hindustani

**Published:** 1846-07

**Authors:** 


Art. IV.—*An Atlas of Anatomical Plates of the Human Body, accompanied with Descriptions in Hindustani.* By Fred. G. Mouatt, m.d., assisted by Moousни Nusseerudin Ahmud.—*Calcutta,* 1846. Fol.

It gives us much pleasure to notice this book, which is printed in Hindustani and edited by Dr. Mouatt. This attempt to diffuse the medical knowledge of the West amongst the inhabitants of the East must meet with the warmest approbation not only of us, who watch over and hail with pleasure the progress of medical knowledge—not only of all well-wishers for the improvement of the Hindoo, but of all philanthropists. This publication is addressed to the military class of the College of Calcutta, and is designed to remove, through the education of the native doctors, so to speak, the ignorance and prejudice of the people. Hitherto Europeans have, for the most part, given their translations of medical works in characters that were not understood by the lower grades; but the editor of the present work has attempted to teach the people by publishing in a language that is understood by them; therefore, the book has been printed in Urdu, "the vernacular language of the people, the vulgar mother-tongue of the mass." From the want of books for the native students in the college, Dr. Mouatt and others have been led to undertake the labour of compiling and translating the following works: a Manual of Anatomy and Physiology, one of Surgery, one of Practice of Medicine and Midwifery, and one of Materia Medica.

The work on anatomy and physiology has been undertaken by Dr. Mouatt, who is assisted by a native, Mooushi Nusseerudin Ahmud. It will consist of an atlas of plates, with accompanying description, which will be followed by a manual of anatomy and physiology; the first part of the Atlas now sent to us from Calcutta, contains drawings of the bones, which are copied from Cheselden's very excellent plates. In the other parts—in all five—it is proposed to give the vessels, the nervous system, the viscera, and organs of sense. There is not any mention made in the preface of illustrations of the muscular system, and the ligaments joining the hard parts of the skeleton. Surely this omission must be from oversight; for what kind of knowledge can be gained of the vessels or nerves without a previous acquaintance with the muscles?

Besides the utility of these works to the native student in medicine, their appearance will be scarcely less beneficial to the junior members of the medical staff in service in India; and we trust that the medical officers of the East India Company's service will use them as a means of familiarizing themselves with the language of the Sepoy committed to their charge. We feel strongly the truth of the remark of the editor, that "no surgeon in charge of a jail or a regiment can be perfectly efficient, or discharge his duties with credit to himself and benefit to his patients, who is entirely ignorant of their tongue, and incapable himself of ascertaining the exact nature and extent of their complaints."

It is because we wish well to this undertaking in the East that we would venture to suggest to Dr. Mouatt the necessity that there will be for his most unremitting attention to the execution of the illustrations. In the progress of a work the artist is so prone, as the novelty wears off, to relax in the ardour necessary to the perfection of the plates. Indeed it is because we notice now some slight disposition to omit the clearness and precision so remarkable in Cheselden's plates that we are induced to offer these remarks. To Dr. Mouatt's name we shall look for security for the fulfilment of the terms of the contract; and we are sure Dr. Mouatt is more desirous of reputation than of the *éclat* of having produced a book.

